# Common Paediatric Elbow Injuries

**DOI:** 10.2174/1874325001711011380

**Published:** 2017-11-30

**Authors:** Christopher E. Hill, Stephen Cooke

**Affiliations:** 1 Specialist Registrar in Trauma and Orthopaedic Surgery, University Hospitals Coventry and Warwickshire NHS Trust, Coventry, UK; 2 Consultant Paediatric Orthopaedic Surgeon, University Hospitals Coventry and Warwickshire NHS Trust, Coventry, UK

**Keywords:** Paediatric elbow, Humeral fractures, Lateral condyle fractures, Medial epicondyle fractures, Radial head, Neck fractures

## Abstract

**Background:**

Paediatric elbow injuries account for a large proportion of childrens’ fractures. Knowledge of common injuries is essential to understanding their assessment and correct management.

**Methods:**

A selective literature search was performed and personal surgical experiences are reported.

**Results:**

We have described the assessment and management of the five most common paediatric elbow injuries: supracondylar humeral fractures; lateral condyle fractures; medial epicondyle fractures; radial head and neck fractures; radial head subluxation.

**Conclusion:**

Understanding of the ossification centres around the paediatric elbow is essential to correctly assessing and managing the common injuries that we have discussed in the review. Outcomes after these injuries are usually favourable with restoration of normal anatomy.

## 1 INTRODUCTION

Childhood fractures are extremely common with a lifetime risk of 42%-64% in boys and 27%-40% in girls [1]. One-third of children will suffer at least one fracture before age seventeen [2] and upper limb fractures account for 72.1% of these [3-5]. Although paediatric elbow fractures are not as common as forearm and wrist fractures, they are of particular importance as they require careful attention to ensure a correct diagnosis and surgical management is more likely to be required to ensure a good outcome [6]. Careful radiographic evaluation is required and an understanding of the ossification centres around the paediatric elbow is essential, (Table **1**)

This review will focus on the five most common paediatric elbow injuries, detailing key points in their treatment from initial presentation, required investigations, injury classification, management options and potential associated complications.

## 2 SUPRACONDYLAR HUMERAL FRACTURES

Supracondylar humeral fractures constitute between 5.5% - 7.7% of all paediatric fractures and over half of elbow paediatric elbow fractures [5, 8, 9] with an estimated annual presentation rate of 177.3 per 100,000 [10]. They occur mostly in children aged 5-8 years old, classically following a fall onto an outstretched hand with the elbow in a position of extension or hyperextension.

### 2.1 Presentation

The child usually presents with a grossly swollen, bruised, painful elbow, often with significant deformity and a refusal to move the affected limb. There may be an associated open injury, or tenting of the skin consistent with an impending open injury. Careful neurovascular examination is critical as 11% will have an associated nerve injury; most commonly the anterior interosseous nerve with extension type injuries, (check with the “OK” sign of flexion of thumb IPJ and index finger DIPJ) or ulnar nerve with flexion type injuries (check with finger abduction and ulnar border little finger sensation), and 1% will have an associated vascular injury [11].

### 2.2 Investigations

**Fig. (1):**
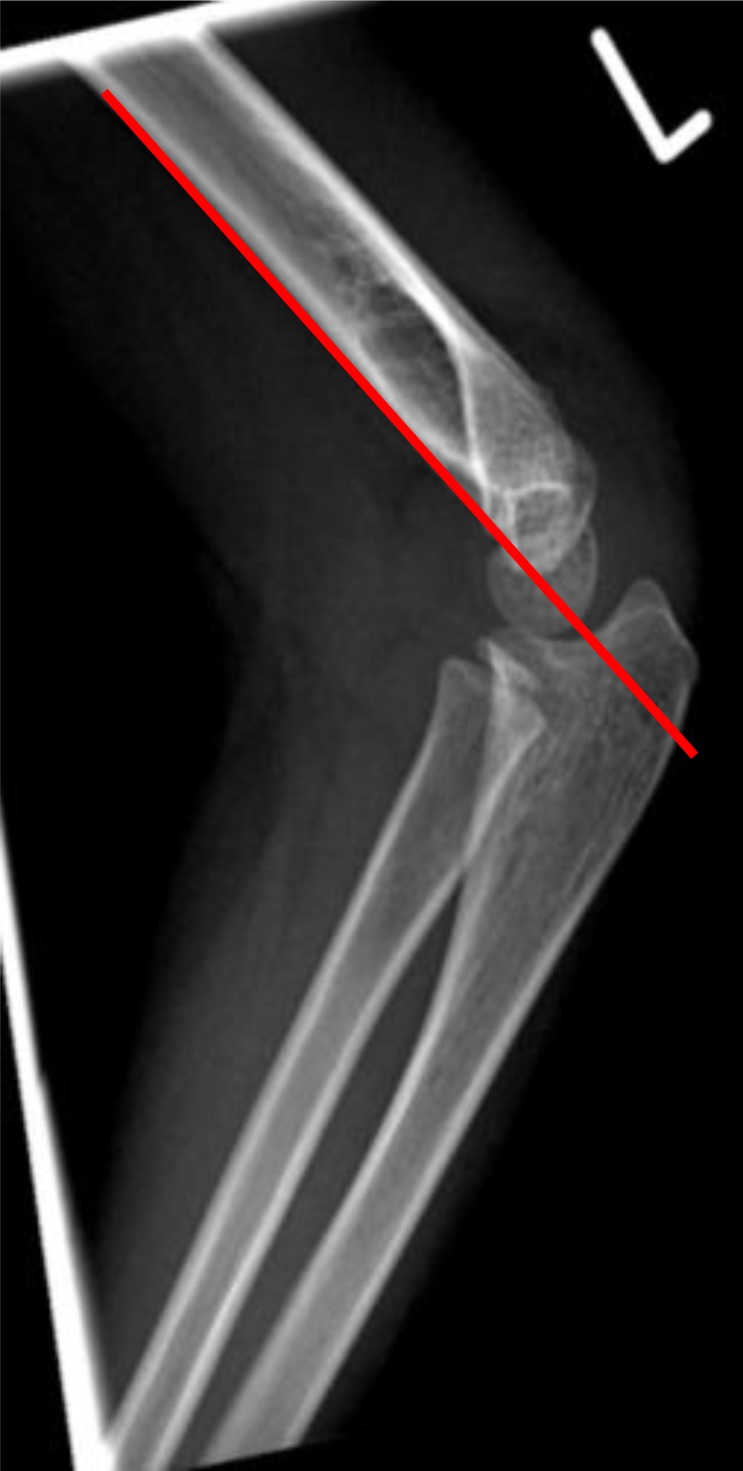
The anterior humeral line intersecting the middle third of the capitellum ossification centre.

**Fig. (2):**
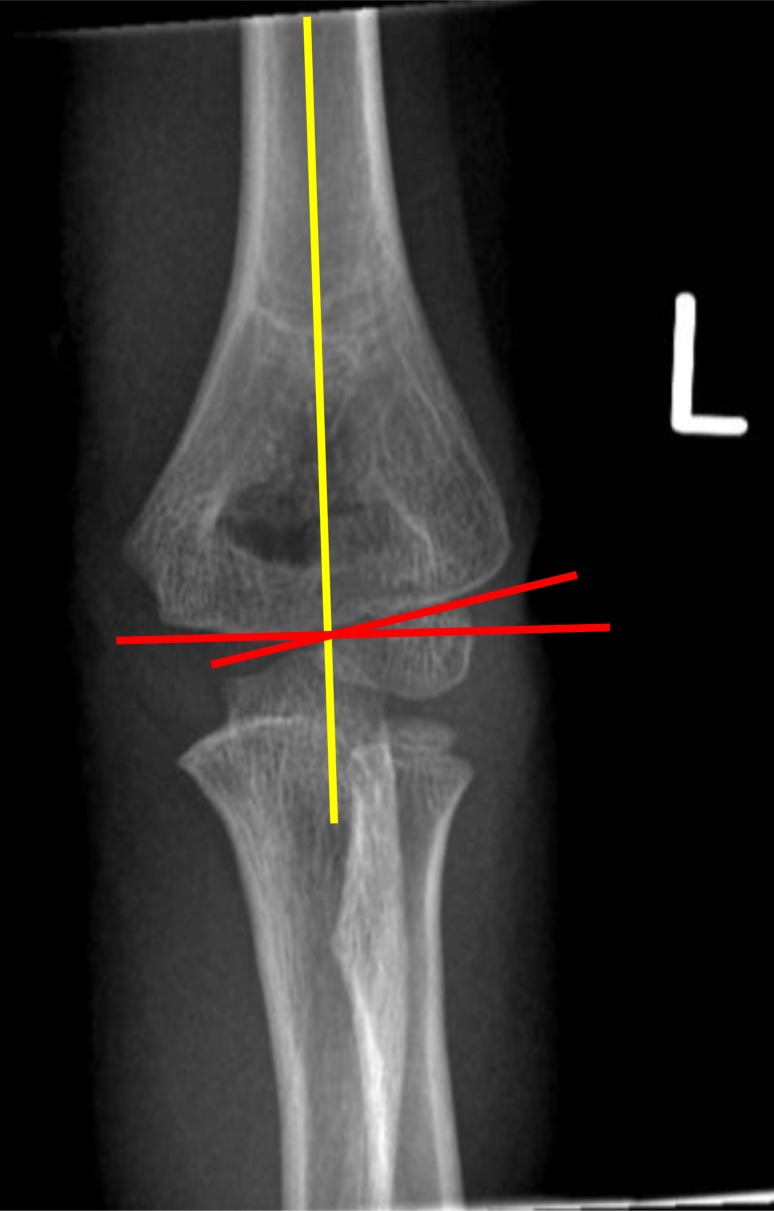
Baumann’s angle, formed by the intersection of a line drawn down the humeral shaft axis and a line drawn along the physeal line of the lateral condyle.

AP and lateral radiographs are usually sufficient to diagnose and classify supracondylar humeral fractures. Assessment of the anterior humeral line (Fig. **1**) (which should intersect the middle third of the capitellum ossification centre) and of Baumann’s angle (Fig. **2**) (the angle formed by the intersection of a line drawn down the humeral shaft axis and a line drawn along the physeal line of the lateral condyle) have been shown to be simple, reliable, repeatable assessments that will reveal the presence of a fracture. The fat pad signs are a useful adjunct in minimally or undisplaced fractures – especially a posterior fat pad which is always pathological [4, 12-15].

### 2.3 Classification

Supracondylar humeral fractures can be broadly classified as either flexion or extension type injuries depending on the direction of fracture displacement. Extension type are by far the most common (>95%) and are traditionally classified using Wilkins Modification of the Gartland classification system [16-18] which classifies injuries by the fracture displacement seen on the initial radiographs. Conservative or surgical management is subsequently recommended based upon the Gartland grade [19], (Table **2**).

### 2.4 Management

Grade 1 injuries with no or minimal displacement are managed non-operatively in a long arm cast in 90-100 degrees of flexion for 3-4 weeks. Grade 2a fractures, with no evidence of rotation, and an anterior humeral line that still intersects any part of the capitellar ossification centre can be managed the same way.

Conservative management of significantly displaced or rotated fractures is usually not advised due to high complication rates [20]. Success with elevated straight arm traction has been shown in some specialist centres [21], but is not widely used and pragmatically speaking, surgical management to reduce and secure fractures anatomically has generally provided superior results compared with non-operative management with both open and closed methods advocated [22-24]. Therefore Grade 2b and 3 injuries are managed surgically with closed reduction and percutaneous pinning +/- open reduction when required (*e.g*. unable to reduce fracture closed, open injury, associated neurovascular injury).

Closed reduction and percutaneous pinning can be achieved using different pin-configurations and is historically a controversial area [25-27]. Early descriptions of percutaneous pinning advocating a medial and lateral cross pin-configuration following closed reduction were described by Swenson [28] and is proffered as being more biomechanically stable than two lateral pins [29]. However, it carries the disadvantage of an increased risk of iatrogenic ulnar nerve injury, estimated to occur in 1 in 28 patients [30], due to the course of the nerve posterior to the medial epicondyle and the fact that the wire is usually placed with the elbow in deep flexion, when in some patients, the ulnar nerve subluxates anteriorly. Thus, an alternative of two pins placed from a lateral position was advocated to avoid injuring the nerve [31]. Although biomechanically less stable, when performed correctly, the technique provides sufficient fixation stability with similar clinical results, without the risk of iatrogenic ulnar nerve injury [11, 32].

Regarding timing of surgery, displaced supracondylar humeral fractures were traditionally considered an orthopaedic emergency requiring immediate operative intervention. This is still the case when neurovascular injury is suspected, or if the fracture is open, however in fractures without significant soft tissue injury or neurovascular compromise, recent evidence has demonstrated no significant difference in peri-operative complications or open reduction rates in children undergoing early versus late surgical treatment. Therefore, it is recommended that surgery should be done on a scheduled trauma list in daylight hours as soon as is safe and practicable to proceed [33-35].

A “**pink, pulseless hand**” on examination presents a further area of controversy as although the pulse is absent, clinically the hand appears warm and pink with a normal capillary refill time and hence is evidently well perfused. Most surgeons recommend urgent closed reduction and pinning followed by a period of close observation rather than openly exploring the injury. In the majority of cases the pulse will return within 24-hours of the injury [36-38]. The simultaneous presence of a nerve injury (usually anterior interosseous or median nerves) has been shown to be strongly predictive of nerve and vessel entrapment; therefore consideration should be given to exploration of the antecubital fossa with vascular surgical support [39].

If the hand is pulseless and poorly perfused urgent closed reduction (within 2 hours) and percutaneous pinning is indicated, as reducing and fixing the fracture may resolve the problem, however if the hand remains poorly perfused then urgent vascular opinion should be sought. The same also applies if the pulse was present pre-operatively but is lost after reduction and pinning given the risk of arterial incarceration in the fracture site. In general arteriography is not warranted at any stage as the location of injury in already known.

### 2.5 Complications

Complications include neurovascular insult sustained at the time of injury but also iatrogenic ulnar nerve injury with medial pin use (3-8%) [30]. Therefore meticulous pre- and post-operative neurovascular examination is essential in these cases. Other complications include pin migration or infection, compartment syndrome – estimated incidence of 1-3 per 1000 fractures [40], with the potential for progression to Volkmann ischaemic contracture (rare), elbow stiffness, which usually resolves with time but end range of elbow flexion and extension may be lost, and angular deformity due to little remodeling potential in the distal humerus, with cubitus varus the most common deformity as a result of malunion, causing a “gunstock” type deformity which is associated with poor cosmesis though minimal impact on function.

## 3 LATERAL CONDYLE FRACTURES

Fractures of the lateral condyle of the humerus are the second most common type of elbow fracture in children with an incidence of 15-17%, typically occurring in children aged 5-10 years old [2, 41]. The mechanism of injury is usually a fall onto an outstretched hand that either transmits a varus force to the distal humerus causing avulsion of the lateral condyle by the common extensor mechanism, or causes impaction of the radial head into the lateral condyle.

### 3.1 Presentation

The child will usually present following a fall with a painful elbow that they are reluctant to use with swelling and tenderness usually limited to the lateral side. The elbow often lacks the degree of deformity seen in displaced supracondylar fractures. Wrist flexion can also exacerbate pain given the pull of the common extensor origin.

### 3.2 Investigations

Imaging should take the form of AP, lateral and oblique radiographs with the internal oblique view often demonstrating the fracture configuration and full extent of displacement [42].

### 3.3 Classification

Classification historically is by the Milch classification [43] (Table **3**) which is dependent on the fracture configuration.

### 3.4 Management

Type 1 fractures can typically be treated with long-arm casting with the forearm supinated for 4-6 weeks. However weekly radiographs for the first two weeks, taken out of plaster are essential, as these fractures have a propensity to displace, thus becoming a type 2 fracture. For this reason some surgeons will justify closed reduction and percutaneous pinning of these injuries in the early stages [45].

Type 2 and type 3 fractures are by definition unstable and require operative management. Most type 2 fractures can be managed with closed reduction and percutaneous pinning with intra-articular reduction confirmed on arthrogram, with divergent pin placement shown to be the most stable configuration [46]. Open reduction is usually required in type 3 fractures where there is a rotational element, in order to ensure intra-articular reduction.

### 3.5 Complications

Lateral condyle fractures are one of the few paediatric fractures that can go on to delayed union or non-union due to the fracture fragment being largely cartilage, bathed in synovial fluid and with a relatively tenuous posterior blood supply which must be protected during any open reduction. Lateral growth arrest can lead to cubitus valgus deformity, which can be associated with tardy ulnar nerve palsy [47].

 Stiffness and loss of end-range extension can also be a problem given the need for a period of immobilization and the intra-articular nature of the fracture. Lateral overgrowth and spurring is another common occurrence (up to 50%) and is correlated with greater initial fracture displacement [48].

## 4 MEDIAL EPICONDYLE FRACTURES

Medial epicondyle fractures, also known as “Little Leaguer’s elbow” due to an association with throwing athletes, most commonly affects children aged 8-14 years, but beware the younger child with a minimally ossified epicondyle. They represent approximately 12% of paediatric elbow injuries [49, 50]. The fracture occurs as a result of traction from the medial collateral ligament and flexor mass avulsing the medial epicondylar apophysis. This usually occurs following a fall on an outstretched hand and is associated with elbow dislocations in up to 50% of cases [49]. This is important as the dislocations will often spontaneously reduce but the medial epicondyle can become incarcerated within the joint in up to 18% of cases and must not be missed [49].

### 4.1 Presentation

Child usually presents with painful elbow following a fall with maximal tenderness focused over the medial epicondylar region. Mild swelling and bruising is usually limited to medial side. Minimal deformity will be present in an isolated injury, however may be present in cases with associated dislocation. Severe pain and swelling with very little or no active or passive movement of the elbow should alert the clinician to the fact that there may be an associated dislocation or incarcerated medial epicondyle.

### 4.2 Investigations

AP, lateral and oblique views are usually sufficient. In a child over 5 years old, the ossific nucleus of the medial epicondyle is usually visible (Table **1**). If it cannot be seen on the AP radiograph, it should be assumed to be incarcerated within the joint until proven otherwise. Careful evaluation of the lateral and oblique views is essential and should be correlated with the clinical findings.

### 4.3 Classification

No real classification system exists other than determining the degree of displacement, and whether or not the fracture fragment is incarcerated within the joint, as these are the key factors for determining management.

### 4.4 Management

The majority of medial epicondyle fractures can be managed non-operatively. As long as the fracture fragment is not incarcerated within the joint, and the displacement is less than 5mm then non-operative management with a long arm cast for 1-2 weeks is appropriate with excellent functional results. Slight controversy revolves around the 5mm of displacement figure, as this is difficult to accurately measure on x-rays and some authors have shown that anywhere between 5-15mm of displacement will heal well by fibrous union without significant symptoms or decreased function.

**Fig. (9):**
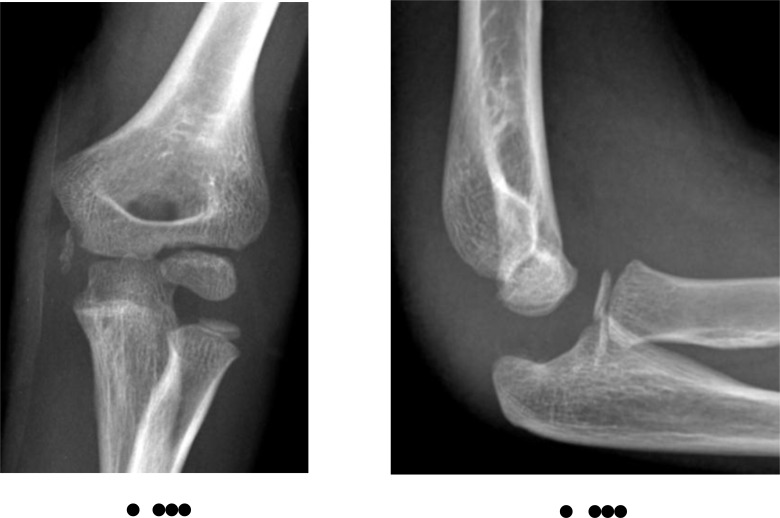
Medial epicondyle fracture with incarceration of fragment within the joint. Note the degree of joint displacement as well as the physical presence of the fracture fragment within.

**Fig. (10):**
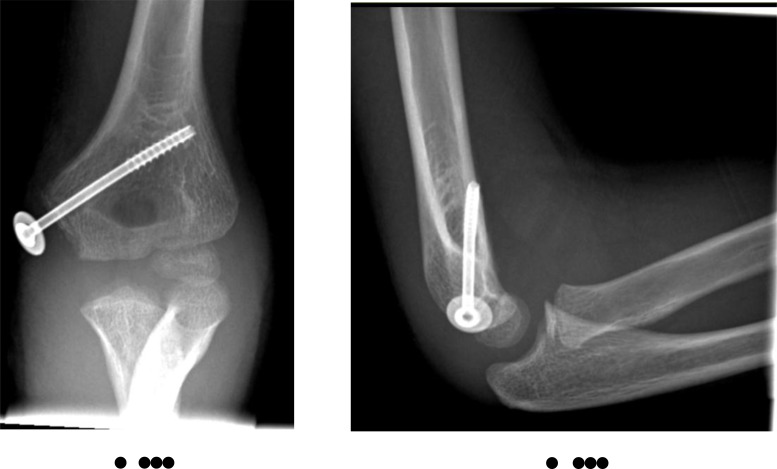
The same patient following open reduction internal fixation of the fracture. Note the size of the “unseen” cartilaginous portion attached to the bony fragment indicated by the washer position to appreciate the full extent of the injury.

Incarceration of the fragment within the joint however is an absolute indication for open reduction and internal fixation, with either wires or cannulated screw fixation, (Figs. **9** and **10**).

Other relative indications for surgery are displacement >5 mm (as discussed above), ulna nerve symptoms (nerve can become entrapped) or displacement in high-level athletes (warranting a faster return to function).

### 4.5 Complications

Complications of these injuries include missed incarceration, ulnar nerve symptoms or injury, stiffness, especially loss of end range extension and non-union – though good functional outcomes reported despite this.

## 5 RADIAL HEAD AND NECK FRACTURES

These injuries make up 5% of all paediatric elbow injuries most commonly affecting a peak age of 9-10 years old [1, 2]. True radial head fractures are relatively rare in the paediatric population, with 90% of these injuries actually being physeal or metaphyseal involving the radial neck. They usually occur following a fall that exerts a valgus force across the elbow and can be associated with elbow dislocations and medial epicondyle fractures.

### 5.1 Presentation

The child with a radial head or neck injury usually presents following a fall with pain and swelling over the lateral side of the elbow, focused maximally in the region of the radial head. They will be reluctant to move the elbow joint, and pronation and supination are especially painful. Referred pain down the forearm is not uncommon.

### 5.2 Investigations

AP and lateral radiographs will often be enough to diagnose and grade these injuries appropriately, however a radiocapitellar (Greenspan [51]) oblique view may be helpful to visualize the extent of the injury more clearly.

### 5.3 Classification

There are various classification systems for these injuries, with on-going controversy as to which is the most helpful. The preferred system of the authors is that of Judet *et al.* [52], which grades the fracture on the degree of displacement, (Table **5**).

Fractures can also be classified by anatomical location or configuration, as in the Wilkins classification [53], (Table **6**).

### 5.4 Management

Fractures with <30**°** of angulation and <50% translation can be managed non-operatively in a long-arm cast for up to 1 week, at which point early mobilization should be performed to prevent elbow stiffness. For fractures with greater displacement, closed reduction under conscious sedation in the Emergency Department may be appropriate depending on the child in order to reduce the fracture sufficiently to proceed with non-operative management. Reduction techniques include:

Patterson technique [54]: Traction with elbow in extension and forearm in supination, then apply varus stress to elbow whilst applying direct pressure over the radial head.

Israeli technique [55]: With the forearm supinated, flex the elbow up to 90**°,** then pronate the forearm with direct pressure over the radial head.

The degree of displacement required for surgical intervention remains controversial, with the best available evidence suggesting in cases where displacement is >45**°** angulation or >50% translation, surgical intervention maybe warranted [56, 57].

This can usually be performed as an assisted closed reduction with a percutaneous wire correction (“joy-stick” technique) or *via* the Metaizeau technique [58] using a TENS nail inserted retrograde up the radial shaft, engaged in the fracture fragment and then rotated to reduce it. Rarely open reduction may be required and should be performed *via* a lateral approach (Kocher-type). This has variable success and a high risk of avascular necrosis of the radial head and of radio-ulnar synostosis however, so should be avoided where possible. Therefore it is the authors’ opinion that following a failed assisted closed reduction, as long as the radial head is providing a strut between the radial metaphysis and capitellum then this will often achieve a superior outcome compared to open reduction internal fixation and should be left, but followed up closely to ensure no further displacement occurs.

### 5.5 Complications

Complications include stiffness with loss of pronation (most common) and supination, radial head overgrowth (20-40%), although this generally has little effect on function, physeal arrest, potentially leading to cubitus valgus deformity, avascular necrosis of the radial head – 10% of fractures, but increases to 70% with open reduction, neurovascular injury, posterior interosseous nerve most commonly affected and radio-ulnar synostosis, associated with open reduction [59].

## 6 RADIAL HEAD SUBLUXATION – “NURSEMAID’s/PULLED ELBOW”

This injury usually affects the younger child with a peak incidence between the ages of 2-5 years old. The mechanism involves traction on an extended elbow causing subluxation and entrapment of the annular ligament over the radial head into the radiocapitellar joint.

### 6.1 Presentation

Parent’s/carer’s will often give a history of “pulling the child along by the hand” or “the child pulled away whilst holding hands” when the child develops sudden pain, begins crying and stops using their arm. The child will often hold their elbow in flexion and pronation, be reluctant to use it and have pain and tenderness localised laterally at the elbow.

### 6.2 Investigations

If the history and clinical picture is appropriate radiographs are not indicated, however if they are performed, AP and lateral radiographs will be normal.

### 6.3 Classification

No classification system exists for this injury.

### 6.4 Management

In acute cases non-operative management is used with closed reduction performed in the Emergency Department. This is achieved by flexing the elbow to 90 degrees, applying gentle pressure over the radial head and progressively pronating and supinating the forearm. Successful reduction is usually confirmed by a return to full movement including pronation and supination and may be accompanied by a satisfying click at the time of reduction. Immobilisation is generally not required and the child can be permitted to continue to use the arm as is comfortable.

### 6.5 Complications

The main complication of note associated with this injury is that of recurrence (5-35%) [60]. This is due to stretching and presence of a tear in the annular ligament at the time of initial injury. Excessive recurrences may warrant operative repair however this is extremely rare, especially as after age 5 further recurrence is uncommon as the distal attachment of the ligament strengthens.

## CONCLUSION

Paediatric elbow injuries are common, and understanding of the ossification centres around the paediatric elbow is essential to correctly assessing and managing the common injuries that we have discussed in this review. With careful and accurate assessment and appropriate management and restoration of normal anatomy however, outcomes after these injuries are usually favourable.

## Figures and Tables

**Fig. (3):**
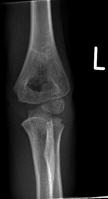
AP radiograph of Wilkin’s-modified Gartland Grade 1 supracondylar humeral fracture.

**Fig. (4):**
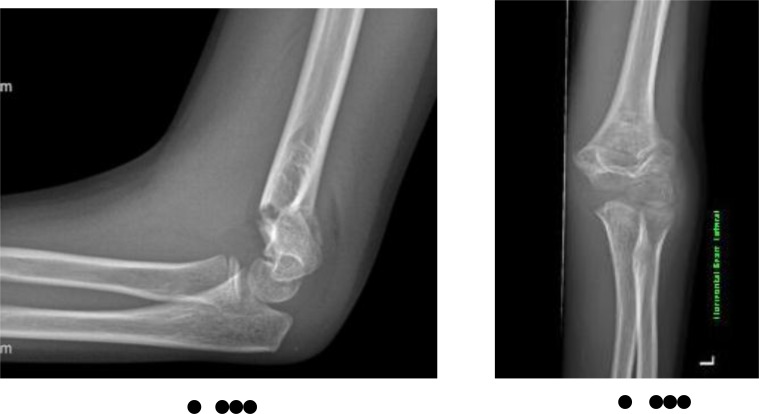
AP and lateral radiographs of Wilkin’s-modified Gartland Grade 2a supracondylar humeral fracture.

**Fig. (5):**
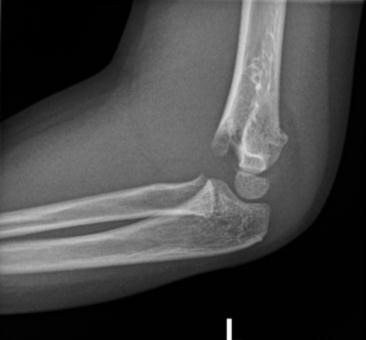
Lateral radiograph of Wilkin’s-modified Gartland Grade 2b supracondylar humeral fracture.

**Fig. (6):**
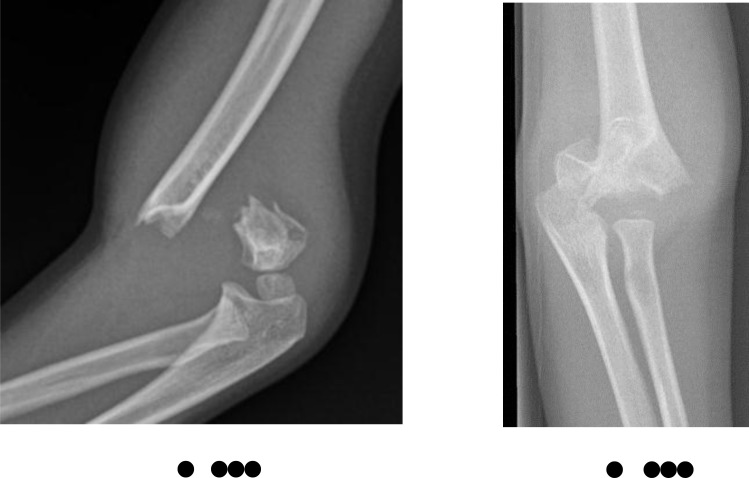
AP and lateral radiographs of Wilkin’s-modified Gartland Grade 3 supracondylar humeral fracture.

**Fig. (7):**
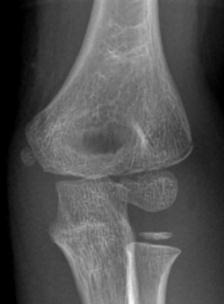
AP radiograph of a Type 1 Jakob Classification lateral condyle fracture.

**Fig. (8):**
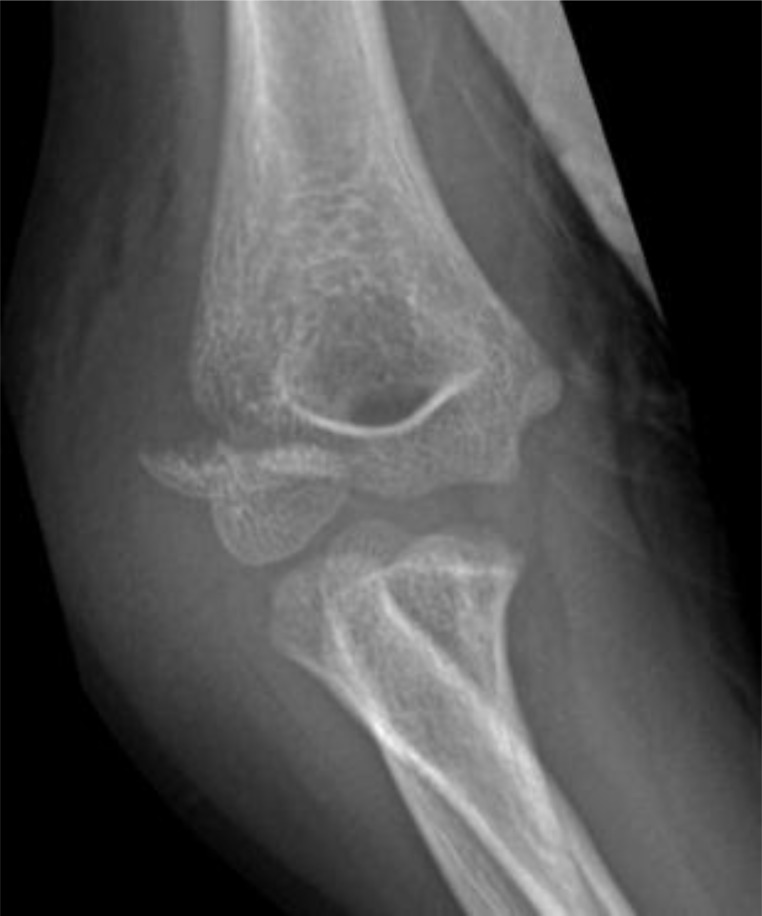
AP radiograph of a Type 3 Jakob Classification lateral condyle fracture.

**Fig. (11):**
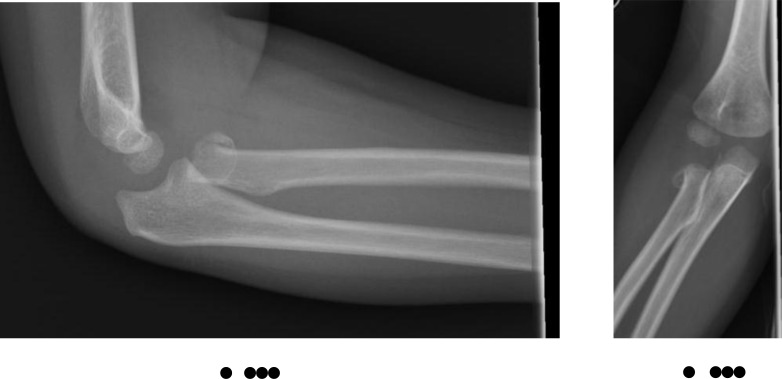
Type IV radial neck fracture as defined by the Judet classification.

**Table 1:** Radiographic evaluation of paediatric elbow – ages at appearance and fusion of ossification centres (+/- 1 year for influence of gender) [7].

Ossification Centre	Age at Ossification Appearance (Years)	Age at Fusion (Years)
Capitellum	1	12
Radius	3	15
Medial Epicondyle	5	17
Trochlea	7	12
Olecranon	9	15
Lateral Epicondyle	11	12

**Table 2:** Wilkins-modified gartland classification system (Figs. **3**-**6**).

**Gartland Grade**	**Fracture Displacement**	**Treatment Method**
Grade 1	No displacement	Conservative
Grade 2a	Angulated in the sagittal plane, but with posterior cortex intact and no translation or rotation	Conservative
Grade 2b	Angulated in the sagittal plane, with rotation	Operative Intervention
Grade 3	Complete displacement	Operative Intervention

**Table 3:** Milch classification of lateral condyle fractures.

Fracture Type	Salter-Harris Equivalent	Fracture Line Extension
Milch Type 1	IV	Fracture line extends through capitellum entering joint lateral to trochlear groove
Milch Type 2	II	Fracture line extends medial to capitellum entering into trochlear groove

However clinically the Jakob classification [44] is much more relevant in guiding treatment (Table **4**).

**Table 4:** Jakob classification of lateral condyle fractures (Figs. **7** and **8**).

Fracture Type	Degree of Fracture Displacement
Type 1	<2mm displacement, intact cartilage epiphyseal hinge
Type 2	2-4mm displacement, joint displaced but not rotated
Type 3	>4mm displacement, joint displaced and rotated

**Table 5:** Judet classification of radial neck fractures (Fig. **11**).

Grade of Injury	Degree of Displacement
I	Undisplaced
II	Angulation <30**°**, Translation <50%
III	Angulation 30**°** – 60**°**, Translation 50-100%
IV	Angulation >60**°**, Translation >100%

**Table 6:** Wilkins classification of radial neck fractures.

Grade of Injury	Fracture location
A	Salter-Harris I or II physeal fractures
B	Salter-Harris III or IV intra-articular fractures
C	Metaphyseal fractures
